# Overdominance at the Gene Expression Level Plays a Critical Role in the Hybrid Root Growth of *Brassica napus*

**DOI:** 10.3390/ijms22179246

**Published:** 2021-08-26

**Authors:** Nesma Shalby, Ibrahim A. A. Mohamed, Jie Xiong, Kaining Hu, Yebitao Yang, Elsayed Nishawy, Bin Yi, Jing Wen, Chaozhi Ma, Jinxiong Shen, Tingdong Fu, Jinxing Tu

**Affiliations:** 1 National Key Laboratory of Crop Genetic Improvement, Hubei Hongshan Laboratory, Huazhong Agricultural University, Wuhan 430070, China; nas05@fayoum.edu.eg (N.S.); xiongjie307@webmail.hzau.edu.cn (J.X.); hukaining@gmail.com (K.H.); yybt1234@163.com (Y.Y.); yibin@mail.hzau.edu.cn (B.Y.); wenjing@mail.hzau.edu.cn (J.W.); yuanbeauty@mail.hzau.edu.cn (C.M.); jxshen@mail.hzau.edu.cn (J.S.); futing@mail.hzau.edu.cn (T.F.); 2 Faculty of Agriculture, Fayoum University, Fayoum 63514, Egypt; iaa04@fayoum.edu.eg; 3 MOA Key Laboratory of Crop Ecophysiology and Farming System in the Middle Reaches of the Yangtze River, College of Plant Science and Technology, Huazhong Agricultural University, Wuhan 430070, China; 4 Desert Research Center, Genetics Resource Department, Egyptian Deserts Gene Bank, Cairo 11735, Egypt; elnishawy@mail.hzau.edu.cn

**Keywords:** heterosis, *Brassica napus*, root growth, overdominant, plant hormones, carbohydrate metabolism, transcription factors

## Abstract

Despite heterosis contributing to genetic improvements in crops, root growth heterosis in rapeseed plants is poorly understood at the molecular level. The current study was performed to discover key differentially expressed genes (DEGs) related to heterosis in two hybrids with contrasting root growth performance (FO; high hybrid and FV; low hybrid) based on analysis of the root heterosis effect. Based on comparative transcriptomic analysis, we believe that the overdominance at the gene expression level plays a critical role in hybrid roots’ early biomass heterosis. Our findings imply that a considerable increase in up-regulation of gene expression underpins heterosis. In the FO hybrid, high expression of DEGs overdominant in the starch/sucrose and galactose metabolic pathways revealed a link between hybrid vigor and root growth. DEGs linked to auxin, cytokinin, brassinosteroids, ethylene, and abscisic acid were also specified, showing that these hormones may enhance mechanisms of root growth and the development in the FO hybrid. Moreover, transcription factors such as MYB, ERF, bHLH, NAC, bZIP, and WRKY are thought to control downstream genes involved in root growth. Overall, this is the first study to provide a better understanding related to the regulation of the molecular mechanism of heterosis, which assists in rapeseed growth and yield improvement.

## 1. Introduction

*Brassica napus* is a main edible oil-producing crop, and for many years, its F_1_ hybrids have been vastly used for commercial purposes [1,2]. Besides using oil rapeseed as edible oil, it can also be utilized as a raw material for bio-energy and many industrial uses [1,3]. In *B. napus*, high growth performance at the seedling stage is pivotal, owing to its contribution to high yield stability and tolerance against abiotic stresses [4,5,6,7,8]. Therefore, the high variation in growth speed enables plant breeders to find key factors related to early growth performance. The high performance of root growth has a crucial role in rapeseed growth, development, and yield formation due to its role in enhancing the production of hormones and amino acids, as well as secreting enzymes, organic acids, and alkaloids to improve the availability of nutrients [9,10]. Agronomically, heterosis expresses high performance in biomass, yield, and tolerance against abiotic and biotic stresses [11]. Several studies have displayed that heterosis levels may be higher in root characteristics than in plant aerial parts [12,13,14]. This implies that roots could be the perfect organ to investigate the genetic basis of *B. napus* heterosis at the early growth stage. Several efforts have been made to expose the molecular system of root growth heterosis during the vegetative stage [15,16,17]. Moreover, previous studies have shown that cotton heterosis can be linked to vegetative heterosis at the seedling stage [18]. Although several genetic models are used to describe heterosis, including dominance and overdominance, the molecular basis remains poorly understood [19,20]. Together with the advancement of functional genomics, many useful technicalities have been used to examine variations in gene expression between parents and hybrids, such as gene expression technology (SAGE) serial analysis, the technology of microarray sequencing, and transcriptome sequencing [21,22,23]. In recent years, numerous transcriptome research progress provided new insights into the molecular foundation of hybrid vigor in *Arabidopsis* [24], rice [8,25], and maize [26] species to explore the mechanism of hybrid vigor at the transcript regulation aspect [27,28]. To better understand heterosis, Thiemann et al. [21] proposed two gene expression-related models: non-additive and additive expression patterns. In diverse crops, genome-wide changes in gene expression have shown varied gene activities (non-additive and additive) as research progresses [26,29,30], implying that the differences in gene dynamics in hybrids are due to the genetic distances between the selected parents [31]. Recently, in hybrid cotton, overdominance was reported to mediate early biomass vigor [4] and play a crucial role in the heterosis of nicotine synthesis and transport efficiency in *Nicotiana tabacum* L. through analysis of the genome-wide comparative transcriptome [32].

Phytohormones are a system of signaling small molecules released by plants that, through gene expression regulation, play an essential function in the growth and development of plants [33,34,35]. Auxin (AUX), one of the most important phytohormones, can regulate primary and lateral root growth by promoting cell division and elongation [36]. Root development is influenced by the expression of genes involved in brassinosteroids (BRs) biosynthesis and signaling [37,38,39]. Moreover, it was reported that there are essential controlling roles for ethylene (Eth) in cell elongation of roots [40], abscisic acid (ABA) in regulating root adaptive responses [34], and cytokinin (CK) in regulating root architecture and nutrient transport proteins [41,42]. However, the increase in the level of CK endogenous level reduces root growth and vice versa [43]. Regarding the crosslink of plant hormones with root growth, gibberellins (GA) interact synergistically with AUX in contrast to CKs, interfering in the regulation of cell expansion and tissue differentiation to induce root elongation and lateral root formation [44,45]. Additionally, evidence has been provided showing the crosslink between CK and BR in promoting the growth of roots [46]. Transcription factors (TFs) control the expression of many related genes involved in the biosynthesis of plant hormone, transport, and signal transduction processes, as well as root growth and development [47,48]. Carbohydrates are important compounds that contribute to the main biological processes necessary for plant growth, in addition to their role as signal molecules [49]. The three main sugars essential to the primary metabolism of plants are sucrose, glucose, and fructose [50,51]. The principal sugar carried through the phloem of most plants is sucrose, which is the end result of photosynthesis. Sucrose is also a source of carbon skeletons, which can be used to make key metabolite components, including starch, cellulose, and proteins [52]. Sucrose is also a crucial signal molecule in plants, coordinating the expression of plant hormones, transcription factors, and other genes [53]. In *Arabidopsis*, sucrose impacts root architecture via regulating the accumulation of endogenous flavonols, which can counteract the effect of ABA on root growth [54]. Many studies have indicated that sugar has an impact on plant root growth [55,56]. Increased glucose concentration leads to increased root length, the number of lateral roots, and root hairs while also modulating the gravitropic response of young seedlings’ primary roots [57].

The molecular basis of early root growth heterosis in *B. napus* was not well documented. Therefore, this study was performed to identify intraspecific DEGs and gene expression as well as pathways of biological processes that mediate heterosis of root growth using two contrasting *B. napus* hybrids and their parents through comparative transcriptome analysis. Furthermore, the obtained data resources may help to discover candidate genes related to biological mechanisms underlying the heterosis of root biomass of rapeseed plants. 

## 2. Results

### 2.1. Morphological Features of Rapeseed Hybrids and Their Parents

**Figure 1:**
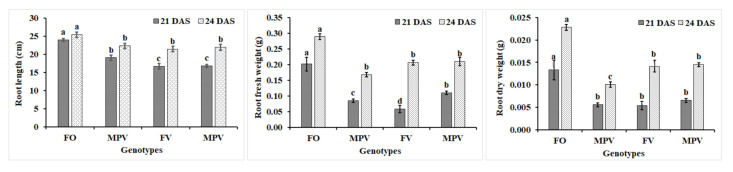
Assessment of the phenotypic parameters (root length, root fresh weight, and root dry weight of two rapeseed F1 hybrids (FO: high hybrid, FV: low hybrid) compared with the mid parent value (MPV) at 21 and 24 DAS. Bars represent the SD of three replicates. Different used letters indicate significant differences (*p* < 0.05) within phenotypic parameters using Tukey’s HSD (Honest Significant Difference) test. FO: High hybrid, FV: Low hybrid, MPV: Mid parent value.

Based on results of the screening of root growth heterosis among rapeseed hybrids, the FO hybrid had the highest root heterosis measurements, while the FV hybrid had the lowest root heterosis measurements. Therefore, they were selected as high hybrid (H) and low hybrid (L), respectively, for further analysis. The phenotypic traits including root length (RL), fresh weight (FW), and dry weight (DW) of roots for the selected hybrids and their three parents (denoted as F, O, and V) were used to confirm the root hybrid vigor over two time points (21 and 24 days after sowing (DAS)) through performing analysis of root heterosis effects. The FO hybrid showed highly significant differences (*p* < 0.05) in RL, FW, and DW as compared with its mid-parent value (MPV) at 21 and 24 DAS (Figure 1). The difference in all root traits between the FV hybrid and its parents was not significant at 21 DAS, while its parents showed substantial differences in FW and DW at 24 DAS. At both 21 and 24 DAS, we observed a significant difference (*p* < 0.05) in the RL, FW, and DW of the FO hybrid as compared with the FV hybrid and the MPV of the FV hybrid (Figure 1).

To measure the heterosis of FO and FV hybrids, mid-parent heterosis (MPH) and high-parent heterosis (HPH) were calculated (Supplementary Table S1). The MPH and HPH of RL, FW, and DW showed a significant difference (*p* < 0.05) between the FO and the FV hybrids. The degree of heterosis for these traits was greater in the FO hybrid than in the FV hybrid. The values of MPH and HPH for all traits in the FO hybrid varied from 14.02% to 140.34% higher than the mid-parent and from 11.32% to 123.02% higher than the high-parent, respectively. While the FV hybrid varied from −0.531 to −46.73 lower than the mid-parent and from −1.81 to −60.11 lower than the high-parent, respectively (Supplementary Table S1). This indicates that the FO hybrid has a high ability to accumulate more biomass in roots compared with its parents and the FV hybrid during the early stage of seedling growth. Therefore, this phenotypic may be a good way to discover the target mechanisms and genetic molecular basis related to root biomass heterosis in rapeseed plants.

### 2.2. RNA-Seq Data Analysis of Different Root Samples

Thirty cDNA libraries were constructed from the total RNA of root samples of the five studied-rapeseed genotypes at 21 and 24 DAS to identify heterotic transcripts of variegated seedlings. After removing sequencing adapters and low-quality data, the sequencing and assembly had 475,213 million clean reads. The mean GC content and Q20 (sequencing error rate = 1%) of all samples were about 46% and 98%, respectively. The output statistics of the transcriptome sequencing are offered in Supplementary File S1. The obtained results elucidated that the Illumina sequencing data was accepted and could be used for further analysis. F1 hybrid gene expression levels were intermediate between parental lines or near one of the parents, implying that mid-parent expression in F1 hybrids can contribute to heterosis [11,58,59]. Therefore, the results obtained from such a comprehensive investigation would extend a confirmatory picture to selecting the target genes associated with root heterosis in rapeseed plants.

**Figure 2:**
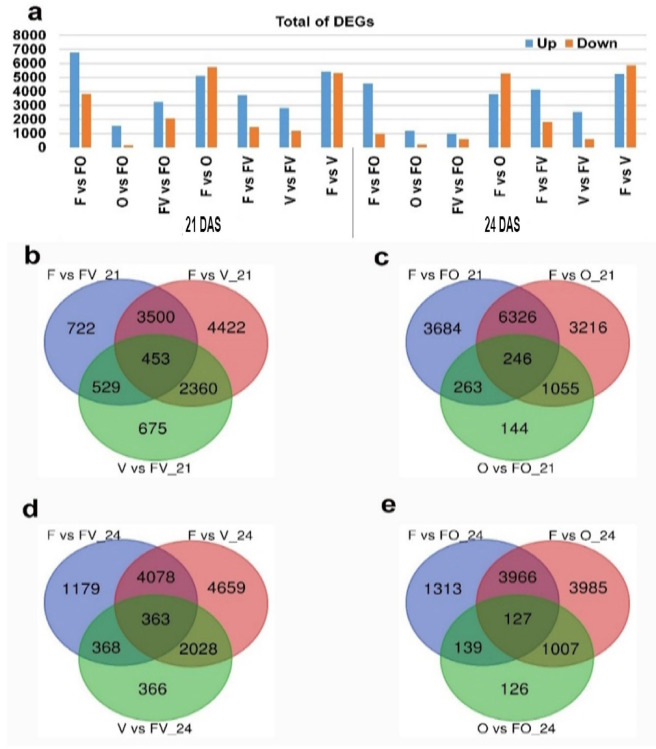
The distribution of differentially expressed genes (DEGs) in root samples of two contrasting hybrids and their parents at 21 and 24 days after sowing (DAS). (**a**) A column chart observes the total number of DEGs (up and down-regulation). (**b**,**d**) Venn diagrams represent distributions of uniquely and commonly DEGs in low hybrid parent triad (FV). (**c**,**e**) Venn diagrams show the distribution of unique and common DEGs in a high hybrid parent triad (FO). O and V: represent maternal parents. F: represents the paternal parent.

To detect the target genes involved in root growth and development, DESeq2 software was used to identify the DEGs between the roots of seedling classes that displayed high (FO hybrid) or low (FV hybrid) root growth. DEGs were selected at both time points (21 and 24 DAS) among different pairwise comparisons of O vs FO, F vs FO, FV vs FO, F vs FV, and V vs FV (Figure 2a, Supplementary Files S2–S5). In each hybrid–parent triad, the levels of expression were significantly different at *p*-adjusted < 0.05 with log2 (fold change) ≥ 1 or log2 (fold change) ≤ −1 considered as DEGs. The total number of up-regulated and down-regulated DEGs among the different pairwise is represented in Figure 2a. In total, 75281 genes were generated (including 5418 novel genes and 69863 known genes). A total of 10607 and 5545 DEGs were significantly detected in the root samples of F vs FO with 6784 and 4564 up-regulated genes and 3823 and 981 down-regulated genes at 21 and 24 DAS, respectively. In O vs FO, 1708 and 1399 DEGs were detected with 1545 and 1185 up-regulated genes and 163 and 214 down-regulated genes at 21 and 24 DAS, respectively. We detected 5331 and 1607 DEGs in FV vs FO with 3257 and 1012 up-regulated genes and 2074 and 595 down-regulated genes at 21 and 24 DAS, respectively. For F vs FV, a total of 5204 and 5988 DEGs were significantly detected with 3741 and 4163 up-regulated genes and 1463 and 1825 down-regulated genes at 21 and 24 DAS, respectively. A total of 4017 and 3125 DEGs were detected in V vs FV with 2834 and 2538 up-regulated genes and 1183 and 587 down-regulated genes at 21 and 24 DAS, respectively (Figure 2a, Supplementary Files S2–S5). We detected higher DEGs in F vs FO compared with O vs FO, indicating that the FO hybrid is close to the O parent than the F parent at both time points. The up-regulated genes were more than down-regulated genes, showing that the root development vigor may be due to the up-regulated genes. A higher number of DEGs was observed in F vs FO than in F vs FV, indicating that the FO hybrid has high root growth vigor than FV at both time points. More DEGs were detected in the F vs O, indicating high root growth vigor among F and O parents (Figure 2a). The Venn diagram analysis revealed that a total of 263 DEGs were commonly observed between F vs FO and O vs FO, whereas 529 DEGs were commonly observed between F vs FV and V vs FV at 21 DAS (Figure 2b,c). A total of 139 DEGs were commonly observed between F vs FO and O vs FO, whereas 368 DEGs were commonly observed between F vs FV and V vs FV (Figure 2d,e).

### 2.3. F_1_ Hybrids Exhibited an Over-Dominant Gene Expression Pattern

**Figure 3:**
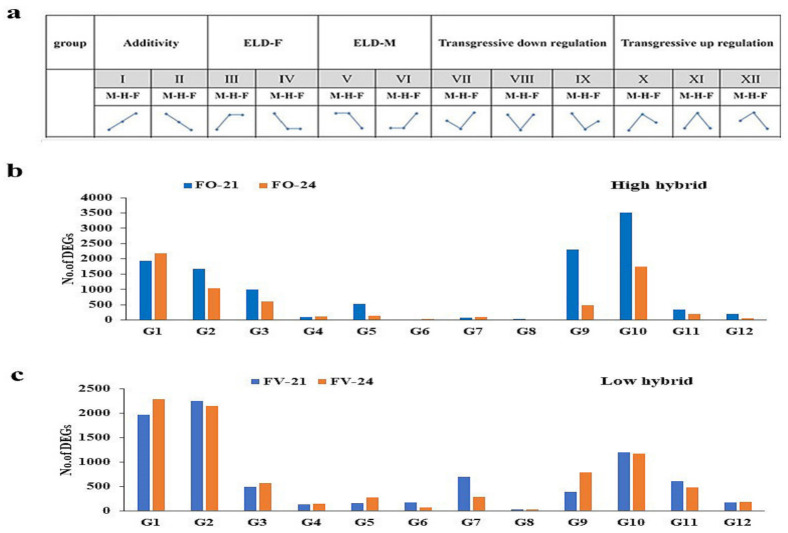
The 12 obtained patterns of gene expression in the root samples of F1 hybrids compared to their parents. (**a**) The pattern of gene expression is divided into 12 groups. M refers to the male parent; H refers to the hybrid; and F refers to the female parent. (**b**,**c**) The distribution of the total number of DEGs into 12 groups (G1:G12) in the roots of the high (FO) and low (FV) hybrids.

To address the directionality and magnitude of expression in interspecific F_1_ *B. napus* hybrids, root transcriptome DEGs were divided into 12 possible classes, as defined by Rapp et al. [60]. The additive expression pattern was observed in groups 1–2 and expression level dominance-female (ELD-F) in groups 3–4, while expression level dominance-male (ELD-M) was observed in groups 5–6. Gene expression patterns in groups 7–9 were mentioned as down-regulated overdominance, while the pattern of gene expression in groups of 10–12 was called up-regulated overdominance (Figure 3a). The expression pattern analysis of both male and female parents had few genes. However, the overdominant groups of up-regulated (10 group) and down-regulated (7 and 9 groups) were found to have the largest number of genes in root tissues (Figure 3b,c, Supplementary Files S6–S9). Therefore, at the gene expression level, our results reveal that overdominance contributes to early root biomass heterosis in rapeseed.

### 2.4. Functional Annotations Gene Ontology (GO) and Kyoto Encyclopedia of Genes and Genomes (KEGG) of Over-Dominant Genes

**Figure 4:**
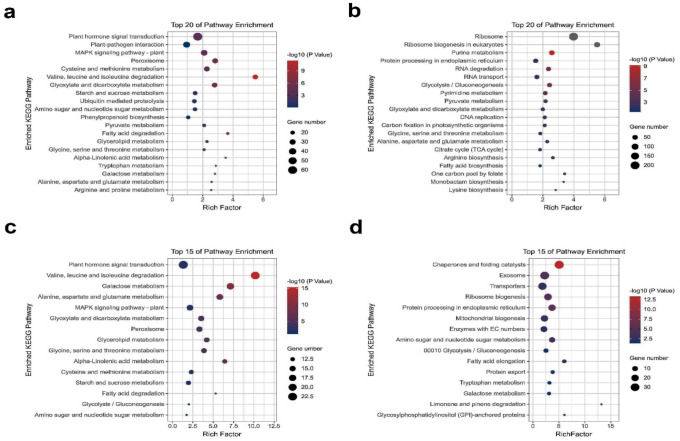
KEGG pathway enrichment analysis for differential gene expressions (DEGs) of high hybrids and its parents. (**a**,**c**) KEGG analysis of DEGs with an up-regulated and down-regulated overdominance pattern at 21 DAS, respectively. (**b**,**d**) KEGG analysis of genes with an up-regulated and down-regulated overdominance pattern at 24 DAS, respectively.

The enrichment analysis by GO of the obtained overdominant genes (*p*-value < 0.05) in FO and FV hybrids relative to their parents revealed that most of the up-regulated and down-regulated genes were involved in “biological regulation”, “cellular process”, “metabolic process”, and “response to stimulus” in the category of biological process (Supplementary Figure S1, Supplementary Files S10–S13). The enriched terms in the category of molecular function were “catalytic activity” and “binding”, while in the category of cellular components were “cell part”, “cell”, and “organelle” (Supplementary Figure S1, Supplementary Files S10–S13). The KEGG pathway enrichment analysis (*p*-value < 0.05) in the FO hybrid relative to its parents at both time points revealed overdominant genes, where most of the up-regulated genes were highly enriched in “plant hormone signal transduction”, “plant–pathogen interaction”, “MAPK signaling pathway”, “peroxisome”, “starch and sucrose metabolism”, and “galactose metabolism” (Figure 4, Supplementary Files S14–S17). However, most down-regulated genes were enriched in “ribosome”, “ribosome biogenesis eukaryotes”, “RNA degradation”, “RNA transport”, and “glycolysis/gluconeogenesis”. Therefore, we selected overdominant DEGs related to plant hormone signal transduction, starch/sucrose, and galactose metabolism pathways for further analysis (Figure 4, Supplementary Files S14–S17).

### 2.5. Role of Carbohydrate Metabolism in Heterosis

**Figure 5:**
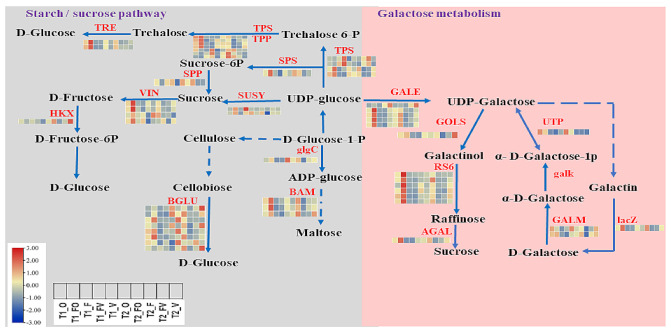
Diagram chart of two carbohydrate metabolism-related pathways for hybrid overdominance genes in the root of rapeseed and their expression. Starch/sucrose and galactose metabolism pathways. The expression profile of DEGs is showed in heatmaps according to counts of the normalized feature (log2 (1 + counts)) using the depth red color represented the up−regulated gene expression and blue represented the down−regulated gene expression. SUSY: Sucrose synthase; SPP: Sucrose phosphate phosphatase; SPS: Sucrose phosphate synthase; VIN; Vacuolar invertase; HKX: Hexokinase; TPS: Trehalose 6−phosphate synthase; TPP: Trehalose 6−phosphate phosphatase; TRE: Trehalase; BGLU: Beta−glucosidase; BAM: Beta−amylase; glgC: Glucose−1−phosphate adenylyltransferase; GALE: UDP−glucose 4−epimerase; UTP: Alpha−D−galactose−1−phosphate uridylyltransferase; GOLS: Inositol 3−alpha−galactosyltransferase; LacZ: Beta−galactosidase; RS6: Raffinose synthase 6; AGAL: Alpha−galactosidase; galK: Galactokinase; GALM: Aldose 1−epimerase. T1: 21DAS; T2: 24 DAS.

Many overdominant DEGs are related to carbohydrate metabolism, including starch/sucrose and galactose metabolism pathways (Supplementary File S18). In the starch/sucrose metabolism pathway, sucrose phosphate synthase (SPS4; *BnaA02g23460D*), vacuolar invertase (VIN; *BnaC09g13230D*, *BnaA09g12880D*, *BnaA09g10560D*, and *BnaA09g10570D)*, and sucrose synthase (SUSY; *BnaA09g00710D*), which catalyze the inverse metabolism, were up-regulated in the FO hybrid (Figure 5). This evidence indicates that the sucrose imports and accumulations in roots may involve its reversal into hexose sugars for use by invertase and sucrose synthase in a variety of ways [61], suggesting that the F1 hybrids produced and utilized more sucrose than their parents. Other enzymes, transcriptional expression levels of beta-glucosidase (BGLU3; *BnaC07g22990D*, *BnaA04g29560D*, BGLU13; *BnaA09g14750D*, *BnaC04g03730D*, BGLU29; *BnaC04g03760D* and *BnaC03g43570D*, and BGLU40; *BnaA08g19770D*), and trehalose 6-phosphate synthase (TPS1; *BnaA07g34230D* and *BnaC06g39000D*, TPS8; *BnaA02g14790D* and *BnaC02g19750D*), trehalose 6-phosphate phosphatase (TPP9; *BnaA08g20280D*, *BnaC08g06450D*, TPP1; *BnaA09g09720D*, *BnaC09g51060D*, TPPA; *BnaAnng22210D*, and TPPB; *BnaA07g33960D* and *BnaC06g38600D*) and trehalase1 (TRE1; *BnaC07g38690D* and *BnaA03g46430D*) were also up-regulated in the FO hybrid (Figure 5). Suggesting that the increase in sucrose level occurred in the high hybrid through the increase in sucrose biosynthesis and accumulation. In the galactose metabolism pathway, aldose 1-epimerase (GALM; *BnaC03g57220D* and *BnaA05g22280D*), UDP-glucose 4-epimerase (GALE; *BnaC08g15730D*, *BnaA01g13540D*, *BnaC05g49280D*, *BnaC01g15760D,* and *BnaA08g24600D*), beta-galactosidase (lacZ; *BnaC04g27780D*), inositol 3-alpha-galactosyltransferase (GOLS2; *BnaC09g14710D*), raffinose synthase6 (RS6; *BnaC09g37530D*, *BnaA02g04780D*, *BnaA09g36810D*, *BnaA10g15160D*, *BnaC09g37510D,* and *BnaC08g28500D*), and alpha-galactosidase2 (AGAL2; *BnaC03g03520D*) were significantly up-regulated in the FO hybrid (Figure 5).

### 2.6. Expression Level of Over-Dominant Hormone Signal Transduction Genes 

**Figure 6:**
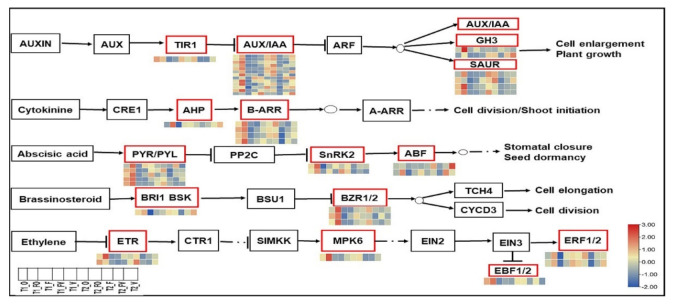
Diagram charts of plant hormone signal transduction pathway supported by heatmap analysis of normalized expression data in *B. napus* hybrids (FO and FV) and their parents. AUX1; Auxin1, TIR1: Transport inhibitor response 1, GH3: Gretchen Hagen 3, AUX/IAA: Auxin/indoleacetic acid, SAUR: Small auxin−up−regulated RNA, CRE1: Cytokinin receptor, AHP: Histidine−containing phosphotransfer protein, B−ARR: Type B−Arabidopsis response regulator, A−ARR: Type A−Arabidopsis response regulator, PYR: Pyrabactin resistance, PYL: PYR−Like, PP2C: Protein phosphatase 2C, SnRK2: Sucrose non−fermenting−1−related protein kinase2, ABF: Abscisic acid−responsive element binding factor, BRI1: Brassinosteroid insensitive1, BSK: BR−Signaling kinase1, BSU1: BRI1 Suppressor1, BZR1/2: Brassinazole resistant 1/2, TCH4: Xyloglucan:xyloglucosyl transferase, CYCD3: Cyclin D3, ETR: Ethylene response, CTR1: Constitutive triple response 1, MPK6: Mitogen−activated protein kinase 6, EIN2: Ethylene-insensitive 2, EIN3: Ethylene insensitive 3, EPF1/2: Epidermal patterning factor 1/2, ERF1/2: Ethylene response factor1/2. T1: 21days after sowing (DAS), T2: 24 DAS.

Combined with the functional analysis, the expression level of overdominant DEGs involved in the plant hormone signal transduction pathway was examined to enhance our understanding of signaling that occurs during morphological changes in root growth heterosis of *B. napus* (Supplementary File S19). In the auxin signal transduction pathway, a total of 20 genes were found to be differentially expressed in the F_1_ hybrids and their parents, among which thirteen DEGs were annotated to Auxin/Indole acetic acid (AUX/IAA; IAA2, IAA8, IAA12, IAA13, IAA16, IAA26, and IAA28), four were annotated to small auxin-up RNA (SAUR; SAUR45, SAUR52, and SAUR59) genes, two annotated to GH3 (Gretchen Hagen 3; GH3.7 and GH3.17) genes, and one encoded to transport inhibitor response 1 (TIR1) protein were highly overdominant up-regulated in the FO hybrid at 21 and 24 DAS (Figure 6, Supplementary File S19). Interestingly, more Aux/IAA genes displayed overdominant high expression in roots of rapeseed hybrids. 

In the BRs signal transduction pathway, four DEGs, including one encoded BR-signaling kinase 1 (BSK1), are hypothesized to regulate BR signal transduction in the root growth. Three brassinazole-resistant 1/2 (BZR1/2) were up-regulated overdominant in the FO hybrid compared to their parents and FV hybrid. These results indicated that high regulation of BR signaling could be considered a crucial factor in improving growth efficiency in the FO hybrid relative to their parents and FV hybrid (Figure 6, Supplementary File S19). In the ABA signal transduction pathway, nine DEGs were identified in the F1 hybrids and their parents, including five abscisic acid receptor PYR/PYL families (PYL1, PYR1, PYL7, and RCAR1), two ABA-responsive element binding factors (ABF including AB15 and ABF3), and two genes encoding sucrose non-fermenting 1-related protein kinase2 (SnRK2 including SnRK2.2 and SnRK2.6), were significantly up-regulated in the FO hybrid compared to the FV hybrid and their parents (Figure 6, Supplementary File S19). These results propose an interesting expression of redundancy within subclass 1 SnRK2 protein kinases, with SNRK2.2 and SNRK2.6 controlling root growth heterosis in rapeseed. In the CK signal transduction pathway, histidine-containing phosphotransfer proteins (AHPs) have a positive regulatory role for CK signaling. It was reported that the expression of one DEG encoding AHP2 and four annotated to type-B response regulators (B-ARRs including B-ARR1, B-ARR2, and B-ARR11) were overdominant up-regulated in the FO hybrid (Figure 6, Supplementary File S19). For the Eth signal transduction pathway, the current results revealed that the negative regulators of Eth signal transduction such as ethylene receptor sensor2 (ERS2; *BnaC03g31730D* and *BnaC08g00620D*) and EIN3-BINDING F-BOX PROTEIN1/2 (EBF1/2 including EBF2; *BnaC05g00620D*), as well as mitogen-activated protein kinase 6 (MAPK6; *BnaA03g26790D*) and ethylene-responsive transcription factor 1/2 (ERF1/2 including ERF15; *BnaC03g17400D* and *BnaC04g13510D*), were overdominant up-regulated in the FO hybrid compared to their parents and FV hybrid (Figure 6, Supplementary File S19). 

### 2.7. Differentially Expressed Transcription Factors Analysis

**Figure 7:**
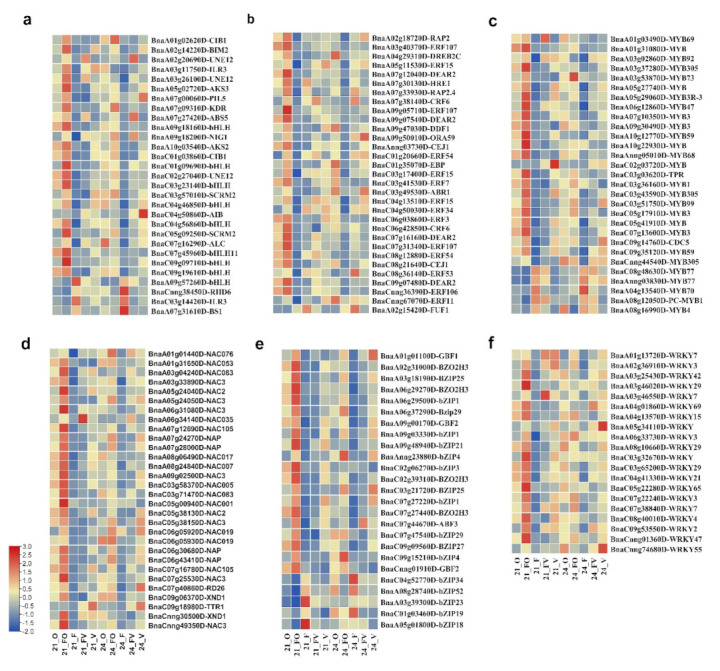
A heatmap illustrating the samples centralized by change expression patterns of differentially expressed transcription factors (TFs) in hybrids (FO and FV) and their parents (O, F, and V). All the TFs were cross−checked with the plant TF database. (**a**) bHLH, (**b**) ERF, (**c**) MYB, (**d**) NAC, (**e**) bZIP, and (**f**) WRKY. The expression profiles of DEGs shown in heatmaps according to counts of the normalized feature (log2 (1 + counts)) using the red depth color representing the expression of up−regulated genes and blue representing the expression of down-regulated genes. T1: 21 days after sowing (DAS); T2: 24 DAS.

Plant transcription factors (TFs) play a role in the regulation of a variety of physiological programs that are crucial for plant life. There were 395 differentially expressed transcripts identified as TFs in our RNA-Seq data (Figure 7, Supplementary File S20). Gene families of TFs showed significantly varied expression between the FO and FV hybrids and their parents for root growth heterosis. TFs belong to the basic helix-loop-helix (bHLH; 29), basic leucine zipper (bZIP; 25), ethylene-responsive factor (ERF; 31), myeloblastosis (MYB; 20), NAC transcription factors (NAC; 30), and W-box containing transcription factor (WARKY; 20), and others were identified as deferentially expressed transcripts. TFs of bHLH, ERF, NAC, and WRKY showed significant up-regulation in the FO hybrid. Moreover, the majority of MYB and bZIP TFs were up-regulated in the FO hybrid compared to the FV hybrid and their parents (Figure 7, Supplementary File S20). 

### 2.8. Validation of RNA-Seq Data

**Figure 8:**
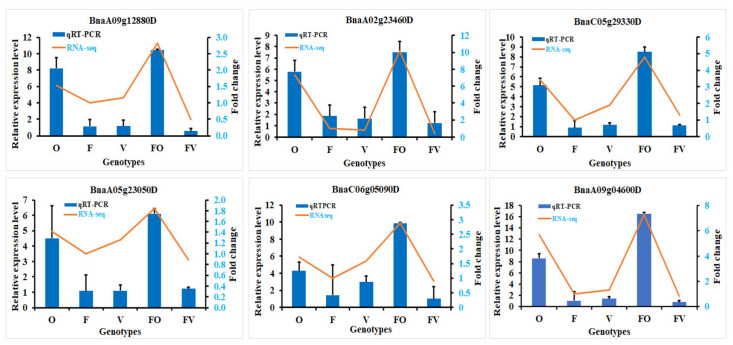
qRT-PCR analysis of randomly selected six differentially expressed genes (DEGs) in all hybrids relative to their parents. FO: High, FV: Low hybrids, and O, F, and V represent three parents. The Actin was used as an internal control in the investigation. Expression patterns transcriptome (RNA-Seq) data for DEGs in FO and FV hybrids as compared to their parent lines (O, F and V) are shown by orange line graphs. Data of qRT-PCR are shown as mean ± SD of three biological replicates.

For the evaluation of the validity of the transcriptome data, we randomly selected six DEGs to carry out a qRT-PCR analysis. The expression levels detected by qRT-PCR analysis of the selected DEGs in the parents and hybrids followed the identical expression as obtained by RNA-Seq (Figure 8). Therefore, these results confirm that our transcriptome data and subsidiary elucidations are reliable.

## 3. Discussion

The heterosis phenomenon refers to the superiority of the offspring over either parent in the desired characteristics or traits. Hybrid vigor breeding successfully utilized the wonders of heterosis to achieve better worldwide crop quantity and quality [62,63]. Hybrid vigor was previously documented during the seedling stage in some plants, such as wheat [47], cotton [4], *Arabidopsis* [64], rice [65], maize [66], and Chinese cabbage [67]. The integration of transcriptome analyses in heterosis studies has effectively provided insights into the molecular basis of heterosis in *Arabidopsis* [24], rice [8,25], maize [26], and easter lily [16], but the molecular basis of root growth heterosis in rapeseed was not documented. During the early stages of growth, high root biomass production plays an important role in improving the absorption of nutrients and water for growth establishment and yield increases in plants [6,68,69]. From this perspective, the genetic basis of root growth and development heterosis was studied in two contrasting hybrids and their inbred parents through comparative transcriptome analysis.

Identifying DEGs between the contrasting hybrids in root biomass heterosis and their inbred parents plays an important role in improving our understanding of hybrid vigor or heterosis [16,32,70,71]. The current transcriptomic data showed that the total number of expressed genes was higher in the FO hybrid than in the FV hybrid and their parents in the root. Therefore, this revealed that the contrasting hybrids had different genomic constituents as compared to their parents. These results predict that the crossing of inbred lines with similar genetics leads to alters in the genetic regulation of the generated hybrids. Due to multiple allelic integrations among two-parent genomes in the cross and reciprocal cross combinations, heterotic genes are robust, indicating that significant genes genetically govern this phenomenon [16]. The expression of DEGs in F_1_ hybrids is statistically classified into 12 classes to determine the gene expression patterns in hybrids relative to parents. These expression patterns indicated an increase in genes of non-additive DEGs in root hybrid tissues. Moreover, up- and down-regulated overdominant DEGs had the greatest number of genes in the FO hybrid, while a few gene numbers were observed in the pattern of ELD-F and ELD-M. Therefore, the overdominant DEGs were used to carry out function analyses to highlight pathways associated with root growth heterosis in rapeseed.

### 3.1. Over-Dominant DEGs Related to Carbohydrate Metabolism Are Involved in Root Biomass Heterosis in Rapeseed Seedlings

Carbohydrate metabolism is a fundamental process in the plant that outputs both structural components and energy of cells [72] and also begets compatible solutes for osmotic adjustment in roots [73,74] that helps to overcome adverse conditions and improves the root system’s ability to uptake water and nutrients [5]. Highly expressed carbohydrate metabolism genes have been documented to improve the heterosis in *Oryza sativa* autotetraploid that contains double neutral genes [49]. In this study, the root tissues of the hybrids and their parents had significant differences in carbohydrate metabolism. This result is consistent with the fact that the root tissues and exudates contain a variety of carbohydrates [75]. The two identified SPS from the DEG analysis were up-regulated in the FO hybrid. The reaction catalyzed by SPS refers to the synthesis of sucrose-6-phosphate (Suc-6-P) from UDP-glucose and fructose-6-phosphate (Fru-6-P), which is considered as an important regulatory step to control sucrose synthesis in many plants [76,77]. Vacuolar invertases (VIN) play a major role in the expansion of plant cells and are considered a key step in the development of plant cells [78], which were up-regulated in the H hybrid. The loss of neutral invertase function can alter sugar metabolism and cause root cell elongation to be reduced. In rice, OsCYT-INV1 is linked to sucrose accumulation and root growth [54]. The high expression or activity of vacuolar invertases has fundamentally been documented in elongating tissues, including sugarcane [79] and *Phyllostachys heterocycle* [80], emphasizing their obvious role in the expansion of plant cells [81,82]. It is involved in *Arabidopsis* seedling hypocotyl cell elongation [81] and rapidly developing tissues in the carrot taproot [83]. Interestingly, the SUS3 gene (*BnaA09g00710D*) was up-regulated in the FO hybrid. SUS1 is primarily found in elongating tissues, such as roots, where secondary wall formation occurs and proceeds rapidly after cell elongation [61,84]. Trehalose 6-phosphate (Tre6P) regulates sucrose levels in plants as both a signal and a homeostatic regulator. Tre6P regulates sucrose synthesis in leaves to maintain a balance between supply and demand for sucrose from increasing sink organs [85]. Trehalose 6-phosphate (T6P) is a critical signaling molecule in plants that regulates sucrose levels both as a signal and as a negative feedback regulator [86]. Some other gene families also involved in a different metabolic pathway related to carbohydrate metabolism are beta-glucosidase (BGLU), UDP-glucose 4-epimerase (GALE), sucrose-phosphate synthase (SPS), and sucrose synthase (SUSY) and were up-regulated in the FO hybrid compared to the FV hybrid and their parents, which is corroborated by the findings of Katara et al. and Zhai et al. [8,22]. Transcriptional expression levels of GALE and AGAL2 were significantly up-regulated in the FO hybrid compared to their parents and the FV hybrid. This reaction is catalyzed by GALE, which catalyzes the conversion of UDP-galactose to UDP-glucose. AGAL1 has been discovered in quiescent center (QC) cells from the meristem of the roots [87,88]. The enzyme UDP-glucuronate 4-epimerase (GALE) catalyzes UDP-glucose into active products that are involved in many other metabolic pathways, including glucuronate and pentose interconversions and nucleotide sugar metabolism [89,90,91].

### 3.2. Overdominance of Hormone Signal Transduction DEGs Are Involved in the Regulation of Root Growth Heterosis of Rapeseed

The growth and development of a plant root system require to be coordinated regulation of endogenous cues as well as environmental signals. Phytohormones are intricately related to plant root growth and development, according to previous research [92]. We found that between F_1_ hybrids and their parents, the overdominant genes encoding hormone signal components were significantly enriched. Aux, CK, ABA, Eth, JA, and BR are plant hormone categories identified, implying that their signaling components are involved in the growth and development of rapeseed roots. Cell division, differentiation, and elongation are directly regulated by Aux during root growth [93]. It was reported that Aux/IAA genes play a critical role in root development [94,95,96]. Through modulation of auxin transportation, SAUR proteins are also involved in proliferation and cell expansion [97,98]. A GH3 family is a significant group of genes related to early auxin-response involved in the development of roots and hypocotyls in *Arabidopsis thaliana* [99]**.** IAA12 protein is instrumental in embryonic primary root formation [100]. CK is a hormone that regulates many developmental processes in plants, including chloroplast formation, root growth, and nutrient uptake [101]. In the root, CK regulates the equipoise between cell differentiation and division, which is important for multicellular organisms’ development [102]. B-ARRs are reported to be positive regulators of the CK signal pathway, whereas A-ARRs are reported to be negative regulators [103]. Nguyen et al. and Zubo et al. [104,105] suggested that the B-ARR gene family members (B-ARR1, B-ARR10, and B-ARR12) play large fundamental roles in the regulation of physiological and transcriptional responses to CK. Eth biosynthesis disruption or signal transduction affects the development of roots, hypocotyls, and seeds [106,107]. The current results revealed the up-regulation of ERS2, EBF1/2, ERF1/2, and MAPK6 in the FO hybrid. This refers to the negative relationship between Eth and root growth of rapeseed hybrids. Therefore, we suggest that the decrease in Eth in FO hybrid may be a potential key mechanism that improves the root growth of rapeseed seedlings. Following Street et al. [108] showed that high levels of Eth significantly inhibit root elongation. The MPK3/MPK6 (*BnaA03g26790D*) gene was reported that loss-of-function mutations caused short root phenotypes [109]. PtaERF3 is involved in the production of adventitious and lateral roots in Populus. Its function is related to the pathway of auxin signaling transduction [110]. ABA is important for root growth regulation, the architecture of the root system, and the adaptive response of roots, including hydrotropism [34]. However, there is no complete understanding of the cellular molecular mechanisms that control the action of core abscisic acid signaling molecules in the root system [34]. ABA signaling is also significant in the mature root, where the majority of mineral and water absorption takes place [111]. PYL1 (*BnaC09g19620D*) and PYR1 (*BnaC07g34880D*), two ABA-responsive genes that are up-regulated in FO hybrid, have been demonstrated to increase ABA-induced seed germination, root growth, and stomatal closure [112]. The up-regulated SNRK2.2 (*BnaC07g31800D*) gene was reported for organizing root development under non-stress conditions and is required in all root tissues [113]. VvPYL1 is thought to be important for root growth and drought resistance [114]. BRs are phytohormones that have a great role in the growth and development of a wide range of plants. Plant hormones called BRs are necessary for growth and development. BRs are involved in numerous aspects of root growth, including meristem size management, root hair production, and lateral root initiation [115]. The roots generated from mutants in the biosynthetic and signaling BRs were reported to be phenotypically short [116]. BR-signaling kinases (BSKs) can activate the transduction cascade of the BRs signals, which mediates root QC (quiescent center) division [117]. The up-regulated DEGs *BnaC03g65340D* (encoding BSK1) in the FO hybrid are suggested for regulating BR signal transduction in the primary root [87]. Collectively, the up- and down-regulation of plant hormone DEGs might have a direct or non-direct roles in the developmental mechanisms involved in root hybrid vigor in *B. napus* plants.

### 3.3. Identification of TFs and Their Role in Heterosis

Due to the lack of reported genes that are regulated by TFs in heterosis studies and their precise functions, this study aimed to identify the target TFs involved in the expression of specific genes that may be important in heterosis. The ethylene-responsive transcription factor plays an important role in *Arabidopsis’* main root elongation [118]. HRE1 (At1g72360.1) and AtERF73/HRE1 (*BnaA07g30130D*), belonging to the *Arabidopsis* AP2/ERF family, regulate meristem cell division of roots in *Arabidopsis*, thus playing a critical role in the development of roots [119]. In *Arabidopsis*, bHLH TFs maintain the balance between differentiation and proliferation of cells, therefore controlling the rate of root growth [120]. Our RNA-seq results revealed that three bHLH DEGs, *BnaA02g20690D*, *BnaA03g26100D*, and *BnaC02g27040D,* were up-regulated in the FO hybrids samples, indicating they may have a key role in root growth heterosis. WRKY proteins play a variety of roles in the simulation of plant growth and development [121]. NAC genes are engaged in a variety of signaling pathways that underpin plant response to environmental stresses and developmental activities. The overexpression of NAC genes, such as ANAC019/055/072, enhanced plant tolerance to drought stress [122]. The MYB and ERF gene families are some of the most numerous transcription factor families discovered in higher plants, and they play a role in the growth and development of plants and allow plants to cope with a variety of stressors [123,124]. Overall, we believe the identified TFs play a key role in the intricate regulation of downstream genes associated with plant hybrid vigor.

## 4. Materials and Methods

### 4.1. Plant Materials

In 2015, our research group crossed 12 selected sterile lines from a population (which was composited with Chinese materials) used as female parents with 12 restorer lines from another population used as male parents based on estimation of general combining ability (GCA) analysis [125,126]. The generated hybrids from this cross were produced at Huazhong Agricultural University. Based on the phenotypic statistical analysis of estimation root heterosis effect (data not published) at 30 DAS of all hybrids and their parents, we selected two hybrids with contrasting stable root growth (FO as High hybrid (H) and FV as Low hybrid (L)) and their parents, i.e., O and V as female parents, and F as a male parent. The time points of 21 and 24 DAS were selected as the most critical time of biomass growth heterosis in rapeseed plants based on phenotypic statistical analysis. The selected hybrids and their parents were grown in a controlled greenhouse (70 ± 5% relative humidity) under a 16/8 h of light/dark cycle at 23 ± 2 °C using a hydroponic culture with Hoagland nutrient solution in a randomized block design with three repeats. Uniform seeds were placed over cotton gauze that floated over a Hoagland’s (a quarter of the strength) solution to provide the nutrients and moisture for seed germination [5]. Then, the uniform 7-day-old seedlings were transplanted in a fresh Hoagland solution (pH 5.8; half-strength). One week later, the nutrient solution was replaced with a completely fresh Hoagland solution (pH 5.8). The growth conditions were performed according to the previously reported articles [127,128]. At 21 and 24 DAS, 30 plants per replicate were collected and immediately washed with distilled water to measure phenotypic data, including root length, root fresh weight, and root dry weight. For root dry weight, root parts were exposed to 105 °C in an oven for half-hour and then dried at 60 °C for 72 h.

### 4.2. Estimation and Testing of Root Heterosis Effects

The root heterosis effects were estimated for root length, root fresh weight, and root dry weight traits for the rapeseed hybrids. For root dry weight, root parts were exposed to 105 °C in an oven for half-hour and then dried at 60 °C for 72 h. We measured root length manually. To calculate the HPH and MPH values, the following formulas were calculated: HPH = ((F1 − HP)/HP) × 100%, and MPH = ((F1 − MP)/ MP) × 100% where F_1_ is the value of the trait observed in the hybrid, HP is the value of the high parent. MP is the average value of two parents. A *t*-test was performed to test the hypothesis. 

### 4.3. RNA Extractions and Illumina Sequencing

For RNA extraction, we collected three biological replicates from root samples of the selected hybrids as well as their parents and immediately frozen them using liquid nitrogen before storing them at −80 °C. From each genotype, the TRIzol method (TIANGEN, Beijing, China) was used to extract the total RNA following the manufacturer’s instructions. To remove any contaminating DNA, RNase-free DNase I (Thermo Scientific, Waltham, MA, USA) was used. A NanoDrop^TM^ spectrophotometer (Thermo Fisher Scientific) and an Agilent 2100 Bioanalyzer (Agilent RNA 6000 Nano Kit, Santa Clara, CA, USA) were used to calculate the concentration and purity of total RNA, respectively. Thereafter, library construction and sequencing were carried out using paired-end sequencing technology by Solution BGI Tech Co., Ltd. (Shenzhen, China) on a system of the Illumina HiSeq 2000 platform.

### 4.4. RNA-Seq Data Analysis and Quality Determinations

The fast QC application v0.11.2 was used to process quality reads from raw RNA-Seq data [129], and the Trimmomatics (0.36.5) tool was used to clean the raw reads from low-quality reads and reads containing adapters [130]. Further, the clean reads of libraries were aligned to the Darmor *B. napus* genome reference [131] by HISAT2 (v2.1.0) [132]. The uniquely and properly mapped reads were selected from SAM files by grep and SAM tools [133]. The raw count of all genes was numbered using the feature counts program [134]. Normalized read counts of genes and DEGs analysis were performed with the DESeq2 R package [135]. The selection of DEGs was performed according to the expression deference of log2 (fold change) ≥1 or log2 (fold change) ≤−1 with statistical significance (adjusted *p*-value < 0.05) by the Ballgown R package [136] between the hybrids and their parents.

### 4.5. Functional Annotation and Pathway Analysis of DEGs

The terms of GO of entire data sets of rapeseed DEGs were performed as described by [137] and annotated to the reference genome using the Blast2GO workflow [138]. We employed the WEGO website tool (http://wego.genomics.org.cn, accessed on 1 August 2021) for producing and illustrating classifications of GO functional terms and the distribution of genes [139]. KEGG annotations were made by the KEGG Automatic Annotation Server (KAAS) [140]. GO and KEGG pathway enrichment analyses were performed on the DEGs using TBtools software (http://cj-chen.github.io/tbtools, accessed on 1 August 2021) with the adjusted *p*-value as a rich factor and the threshold [141]. KEGG pathways were assigned to the detected genes in the KEGG database (https://www.genome.jp/kegg/, accessed on 1 August 2021).

### 4.6. qRT-PCR Validation

For the validation of the transcriptome data, we randomly selected six DEGs for qRT-PCR (PCR quantitative real-time) analysis. The software of the Primer Premier 5 application was used for designing specific primers of the selected genes based on the sequence of the reference genome and are listed in Supplementary File S21. The same samples of the RNA-seq library construction were used for qRT-PCR analysis. qRT-PCR was performed using SYBR Green Master Mix Real-Time PCR (Toyobo, Japan) and the system of CFX96 Real-Time (Bio-Rad, Foster, CA, USA). The method of 2^−ΔΔ^CT was used to measure the relative expression level of the selected DEGs [142]. 

### 4.7. Experimental Design and Statistical Analysis

The plant genotypes used in this study were arranged in a randomized complete block design, replicated three times. One-way analysis of variance (ANOVA) was used to determine the phenotypic parameters using the statistical software GenStat 17th edition [143], while Tukey’s Honest Significant Difference (HSD) test at a 5% probability level was applied to distinguish significant levels.

## 5. Conclusions

Root systems enable the uptake of water and nutrient elements, which are pivotal components of plant growth performance and production. For heterosis breeding of *Brassica* crops, hybrid development has been widely used. In the present study, a comparative transcriptome analysis study was performed on the hybrids and their parents. Overall, we discovered that heterosis is linked to an increase in global gene expression. Several DEGs encoding for plant hormones, transcription factors, starch/sucrose, and galactose metabolism responsive genes were identified. Here, we suggest that the identified DEGs may contribute to root biomass heterosis in *B. napus*. The expression levels of most identified DEGs were overdominant in combinations, strongly suggesting that expression level overdominance DEGs have a role in the hybrid vigor of *B. napus*. Therefore, this comprehensive study gives new insights for understanding the molecular basis of mechanisms related to root growth heterosis of rapeseed. The current large-scale data will be a potential source for further molecular studies on seedling growth performance for improving rapeseed production.

## Data Availability

Data is contained within the article.

## Supplementary Materials

The following are available online at https://www.mdpi.com/article/10.3390/ijms22179246/s1, Table S1: Comparison of phenotypic traits by analysis of heterosis measurements for FO and FV hybrids with their parents. Figure S1: Functional annotation of all of the overdominant DEGs in the root of F1 hybrids based on GO classification. File S1: Brief detail of clean data after RNA sequencing and its quality, File S2: DEGs information of roots in the FO hybrid–parent triad at 21 DAS (T1), File S3: DEGs information of roots in the FO hybrid–parent triad at 24 DAS (T2), File 4 DEGs information of roots in the FV hybrid–parent triad at 21 DAS (T1), File S5: DEGs information of roots in the FV hybrid–parent triad at 24 DAS (T2), File S6: Gene expression level data of the roots of FO hybrid as compared to its parents at 21 DAS (T1), File S7: Gene expression level data of the roots of FO hybrid as compared to its parents at 24 DAS (T2), File S8: Gene expression level data of the roots of the FV hybrid as compared to its parents 21 DAS (T1), File S9: Gene expression level data of the roots of the FV hybrid as compared to its parents 24 DAS (T2), File S10: GO enrichment terms of obtained overdominant genes of roots of the FO hybrid–parent triad at 21 DAS, File S11: GO enrichment terms of obtained overdominant genes of roots of the FV hybrid–parent triad at 24 DAS, File S12: GO enrichment terms of obtained overdominant genes of roots of the FV hybrid–parent triad at 21 DAS, File S13: GO enrichment terms of obtained overdominant genes of roots of the FV hybrid–parent triad at 24 DAS, File S14: KEGG enrichment terms of obtained overdominant genes of roots of the FO hybrid–parent triad 21 DAS, File S15: KEGG enrichment terms of obtained overdominant genes of roots of the FO hybrid–parent triad at 24DAS, File S16: KEGG enrichment terms of obtained overdominant genes of roots of the FV hybrid–parent triad at 21 DAS, File S17: KEGG enrichment terms of obtained overdominant genes of roots of the FV hybrid–parent triad 24 DAS, File S18: The dynamic changes in differentially expressed genes (DEGs) of carbohydrate metabolism-related pathways in two contrasting hybrids and their parents, File S19: The dynamic changes in differentially expressed genes (DEGs) of plant hormone signal transduction pathways in two contrasting hybrids and their parents, File S20: The dynamic changes in differentially expressed genes (DEGs) of transcription factors (TFs) families in two contrasting hybrids and their parents. File S21: List of designed selected primers used for qRT-PCR analysis.
