# Supplementary material for: Overdominance at the Gene Expression Level Plays a Critical Role in the Hybrid Root Growth of Brassica napus
Source: Int J Mol Sci. 2021 Aug 26;22(17):9246. doi: 10.3390/ijms22179246 (PMC8431428; doi:10.3390/ijms22179246)
Supplement: Supplementary file 1 [file ijms-22-09246-s001.zip › ijms-1346590-supplementary/Supplementary Materials/Supplementary Figure S1 .pdf]

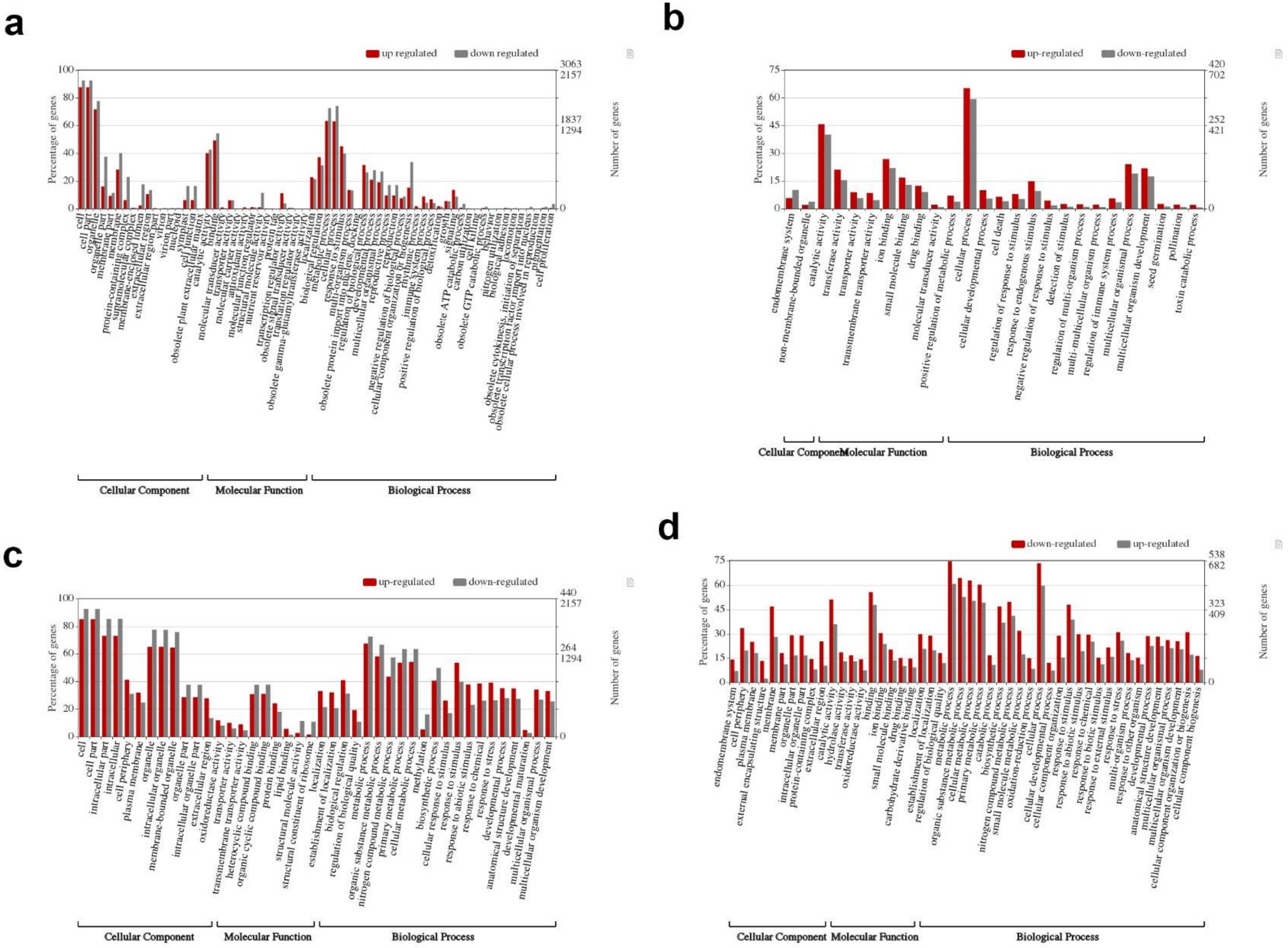
a
up regulated
down regulated
Percentage of genes
Number of genes
100
80
60
40
20
0
3063
2157
1837
1294
Cellular Component
Molecular Function
Biological Process
b
up-regulated
down-regulated
Percentage of genes
Number of genes
75
60
45
30
15
0
420
702
252
421
endomembrane system
non-membrane-bounded organelle
catalytic activity
transferase activity
transporter activity
transmembrane transporter activity
ion binding
small molecule binding
drug binding
molecular transducer activity
positive regulation of metabolic process
cellular process
cellular developmental process
cell death
regulation of response to stimulus
response to endogenous stimulus
negative regulation of response to stimulus
detection of stimulus
regulation of multi-organism process
multi-multicellular organism process
regulation of immune system process
multicellular organismal process
multicellular organism development
seed germination
pollination
toxin catabolic process
Cellular Componen
Molecular Function
Biological Process
c
up-regulated
down-regulated
Percentage of genes
Number of genes
100
80
60
40
20
0
440
2157
264
1294
cell
cell part
intracellular part
intracellular
cell periphery
plasma membrane
organelle
intracellular organelle
membrane-bounded organelle
organelle part
intracellular organelle part
extracellular region
oxidoreductase activity
transporter activity
transmembrane transporter activity
heterocyclic compound binding
organic cyclic compound binding
protein binding
lipid binding
structural molecule activity
structural constituent of ribosome
localization
establishment of localization
biological regulation
regulation of biological quality
metabolic process
organic substance metabolic process
nitrogen compound metabolic process
primary metabolic process
cellular metabolic process
methylation
biosynthetic process
cellular response to stimulus
response to stimulus
response to abiotic stimulus
response to chemical
response to stress
developmental process
anatomical structure development
developmental maturation
multicellular organismal process
multicellular organism development
Cellular Component
Molecular Function
Biological Process
d
down-regulated
up-regulated
Percentage of genes
Number of genes
75
60
45
30
15
0
538
682
323
409
endomembrane system
cell periphery
plasma membrane
external encapsulating structure
membrane
membrane part
organelle part
intracellular organelle part
protein-containing complex
extracellular region
catalytic activity
hydrolase activity
transferase activity
oxidoreductase activity
binding
ion binding
small molecule binding
drug binding
carbohydrate derivative binding
localization
establishment of localization
regulation of biological quality
metabolic process
organic substance metabolic process
cellular metabolic process
primary metabolic process
catabolic process
biosynthetic process
nitrogen compound metabolic process
small molecule metabolic process
oxidation-reduction process
cellular process
cellular developmental process
cellular component organization
response to stimulus
response to abiotic stimulus
response to chemical
response to biotic stimulus
response to external stimulus
response to stress
multi-organism process
response to other organism
developmental process
anatomical structure development
multicellular organismal process
multicellular organism development
cellular component organization or biogenesis
cellular component biogenesis
Cellular Component
Molecular Function
Biological Process

Supplementary Figure S1 Functional annotation of all of the overdominant DEGs in the root of F_1 hybrids based on GO classification. GO term classification for DEGs was performed by WEGO software. (**a)** The FO hybrid at 21 days after sowing. (**b)** the FV hybrid at 21 days after sowing. (**c)** The FO hybrid at 24 days after sowing. (**d)** The FV hybrid at 24 days after sowing. The obtained results are summarized under three top-level ontologies: biological process, molecular function, and cellular component. The left y-axis indicates the percentage of a specific GO category in that main category. The right y-axis indicates the annotated gene number expressed in a given sub-category. The down-regulated DEGs are represented by grey, and the up-regulated DEGs are represented by red.
